# A Systematic Review and Dose-Response Meta-Analysis of the Association Between the Age of First Pregnancy and Breast Cancer Risk

**DOI:** 10.34172/jrhs.13923

**Published:** 2026-08-15

**Authors:** Jalal Poorolajal, Fatemeh Shahbazi, Nazanin Azmi-Naei, Amin Doosti-Irani, Bita Azmi-Naei

**Affiliations:** ^1^Department of Epidemiology, School of Public Health, Hamadan University of Medical Sciences, Hamadan, Iran; ^2^Modeling of Noncommunicable Diseases Research Center, Institute of Health Sciences and Technologies, Hamadan University of Medical Sciences, Hamadan, Iran; ^3^Research Center for Health Sciences, Institute of Health Sciences and Technologies, Hamadan University of Medical Sciences, Hamadan, Iran; ^4^Department of Epidemiology, School of Public Health, Shahroud University of Medical Sciences, Shahroud, Iran

**Keywords:** Breast neoplasms, Pregnancy, Age groups, Dose-response relationship, Systematic review, Meta-analysis

## Abstract

**Introduction:**

Despite numerous investigations, variations in breast cancer (BC) risk across different ages at first pregnancy remain inconclusive. Thus, this meta-analysis aimed to clarify this association and provide a comprehensive evaluation of the evidence.

**Study Design:**

A systematic review.

**Methods:**

PubMed, Web of Science, and Scopus databases were systematically searched to identify observational studies published up to March 16, 2025, examining BC risk in relation to age at first pregnancy. Statistical analyses were performed using Stata 18 and Review Manager 5.4. Heterogeneity and potential sources were assessed using the I^2^ statistic and meta-regression, respectively. Publication bias was also evaluated with Begg’s and Egger’s tests. Pooled effect sizes were expressed as risk ratios (RR) or odds ratios (OR) with 95% confidence intervals (CI) using a random-effects model.

**Results:**

Of 15,586 identified records, 141 studies involving 1,045,983 participants met the inclusion criteria. The pooled RR of BC for age at first pregnancy≥20 years versus<20 years was 1.18 (95% CI: 1.09–1.28), and the pooled OR was 1.31 (95% CI: 1.26–1.37). Moreover, dose-response meta-analysis demonstrated a consistent upward trend, indicating that BC risk increases by 8–12% for every 5-year delay in the first pregnancy.

**Conclusion:**

Our findings revealed that BC risk increases progressively with older age at first pregnancy. However, the overall evidence was moderate due to study limitations. Accordingly, further research is warranted to clarify underlying mechanisms and population-specific variations.

## Introduction

 Understanding the association between the age of the first pregnancy and breast cancer (BC) risk is of paramount importance in the field of oncology.^1^ BC is a globally serious health concern due to its high prevalence, impact on morbidity and mortality, and the associated healthcare burden.^2^ It is the most common cancer among women worldwide^2^ and a leading cause of cancer-related deaths in women. It is estimated that over 600,000 women die from BC annually.^3^

 Hence, identifying the modifiable risk factors of BC is crucial for effective prevention and early detection strategies.^1,4^ Epidemiological studies have suggested that the age at which a woman experiences her first pregnancy may play a pivotal role in BC development, as reproductive factors have been shown to influence breast tissue biology and hormonal exposures.^5^ However, the existing literature presents conflicting findings; while some studies report an increased risk associated with early pregnancy,^6-10^ others suggest a protective effect.^11-14^ These inconsistencies highlight the need for a comprehensive analysis to clarify the true relationship between the age of first pregnancy and BC risk.

 It should be noted that previous studies have been limited by small sample sizes, variations in study design, and the inclusion of confounding factors, making it challenging to draw definitive conclusions. Additionally, most studies have focused on comparing early pregnancies with pregnancies at later ages, neglecting the potential differences in risk among different age groups. Accordingly, a dose-response meta-analysis is necessary to synthesize the available evidence and quantitatively evaluate the relationship between the age of first pregnancy and BC risk across various age categories, allowing for a more nuanced understanding of the association and enabling the identification of critical periods during reproductive life that may impact BC risk. Therefore, this systematic review aims to conduct a dose-response meta-analysis to compare the risk of BC among women who experienced their first pregnancy at different age groups.

## Methods

###  Eligibility Criteria (Population, Intervention/Exposure, Control, Outcomes, and Studies)

*Population*: Women of reproductive age were enrolled in the study, with no exclusions based on race, ethnicity, or nationality.

*Intervention/Exposure*: The exposure group included women who had their initial pregnancy at the age of 20 or older.

*Control*: The control group comprised women who experienced adolescent pregnancies, defined as those occurring in females aged 10–19 years.^15^

*Outcome*: The primary outcome of interest was the occurrence of BC confirmed via pathological examination, regardless of tumor type or stage.

*Studies*: Observational studies, including cohort and case-control designs, were considered in the analysis irrespective of publication status or language. These studies specifically examined the association between age at first pregnancy and the risk of BC. To qualify for inclusion, studies had to report effect sizes as odds ratios (OR)/risk ratios (RR) with their corresponding 95% confidence intervals (CI), or provide sufficient data to allow manual calculation of the effect sizes.

 Studies classified as case reports, reviews, or editorials, as well as those lacking adequate information on age at first pregnancy, BC incidence, or other pertinent variables, were excluded from the investigation. To ensure meaningful comparisons, studies that did not directly contrast the 10–19 age group with other age groups in terms of age at first pregnancy were excluded as well.

###  Information Sources and Search Strategy 

 A systematic search was performed in the PubMed, Web of Science, and Scopus databases up to March 16, 2025. In addition, the reference lists of the included studies were examined to identify any further studies meeting the inclusion criteria. Keywords used for search included (breast) AND (carcinoma OR tumor OR malignancy OR neoplasm OR cancer) AND (parity OR pregnant OR pregnancies OR pregnancy) AND (early OR adolescence OR adolescent OR teenager OR teen OR teenage OR youth OR young). It should be noted that keywords were searched both as MeSH terms and text words in order to ensure comprehensive coverage.

###  Selection Process

 The search results from all databases were compiled using EndNote software, and duplicate records were removed. Two authors (Nazanin Azmi-Naei and Bita Azmi-Naei) independently screened the titles and abstracts of the retrieved articles to exclude studies that did not meet the inclusion criteria. In addition, any discrepancies between the authors were resolved through discussion. The level of agreement between the two reviewers was substantial (Kappa = 0.78). Finally, the full texts of potentially relevant studies were obtained and thoroughly assessed to determine their eligibility for inclusion in the analysis.

###  Data Collection Process

 Data from the included studies were extracted by two authors (Jalal Poorolajal and Bita Azmi-Naei) and recorded in an electronic data sheet created using Stata software (version 18). The extracted information covered various parameters, including the first author’s surname, publication year, country, language, age (mean or range), study design (cohort or case-control), age at first pregnancy, sample size, adjustment for potential confounders (adjusted or unadjusted), and effect sizes (RR and OR) with their corresponding 95% CIs. Fully adjusted effect sizes reported by the studies were used whenever available. Adjustments were made for race, ethnicity, age at menarche, menopausal status, educational level, residential area, monthly income, family history of BC, body mass index, hormone replacement therapy, oral contraceptive use, physical activity, alcohol consumption, and cigarette smoking (for one or more of these factors).

###  Study Risk of Bias Assessment

 The methodological quality of the studies was evaluated using the Newcastle-Ottawa Scale (NOS)^16^ focusing on (1) the selection of study groups, (2) the comparability of groups, and (3) the ascertainment of exposure or outcomes. These three key domains aim to assess how representative and comparable the groups are, evaluate the extent to which studies controlled for confounding variables, and examine the reliability of outcome measurements, respectively. Each study could receive a maximum of nine stars, with scores seven or more showing high-quality studies. Studies scoring fewer than seven stars were classified as low-quality, indicating potential biases in study design or outcome assessment.

###  Heterogeneity and Publication Bias Assessment

 Both the chi-square test^17^ and the I^2^ statistic^18^ were employed to assess potential heterogeneity among the studies included in the analysis. The I^2^ value was used to quantify the degree of heterogeneity, with values below 50%, 50–74%, and 75% or higher indicating low, moderate, and high heterogeneity, respectively.^18^ Additionally, meta-regression was conducted to explore potential factors contributing to the observed heterogeneity.

 To evaluate the potential presence of publication bias, both the Egger’s^19^ and the Begg’s^20^ tests were applied, alongside the “Trim and Fill” analysis^21^, which is a statistical approach that addresses publication bias by estimating the number and outcomes of potentially missing studies.

###  Sensitivity Analysis

 In instances of moderate-to-high between-study heterogeneity (I^2^ ≥ 50%), sensitivity analyses were performed using the MetaPlot tool in Stata (version 18).^22,23^ This instrument is based on the sequential method^24^ and provides a graphical approach for identifying studies that substantially contribute to overall heterogeneity. The sequential algorithm in MetaPlot conducts iterative “one-out” sensitivity analyses, excluding one study at a time to assess the impact of individual studies on the overall meta-analysis results. Moreover, this approach offers a clear and efficient means of evaluating the influence of single studies on the overall findings.

 An additional sensitivity analysis was performed to evaluate the influence of high-quality studies compared to low-quality ones on the overall effect.

###  Effect Measures

 In this study, the primary effect measures were RRs and ORs for cohort and case-control studies, respectively. These effect measures were pooled, and the overall summary estimate was reported as either RR or OR using a random-effects model.^25^ Data were analyzed at a significance level of 0.05 using Stata (version 18, Stata Corporation, College Station, TX, USA) and Review Manager software (version 5.4).

 For the dose-response meta-analysis, age at first pregnancy was categorized into five groups: < 20, 20–24, 25–29, 30–35, and > 35 years. The risk of BC in each age group was then compared with that of the reference group ( < 20 years). This approach enabled the assessment of how BC risk changes with increasing age at first pregnancy, using the dose-response command for meta-analysis in Stata software.

 Studies that did not use the five-year categorization but investigated the relationship between age at first pregnancy and BC risk in alternative ways were included to avoid excluding the relevant ones. The results from these studies were analyzed and reported separately, with age under 20 years serving as the reference category.

###  Certainty Assessment

 Following the evidence synthesis, the Grading of Recommendations, Assessment, Development, and Evaluation (GRADE) approach^26,27^ was applied to assess the certainty of the evidence for the outcome. This framework evaluates five domains that may decrease certainty (i.e., risk of bias, inconsistency, indirectness, imprecision, and publication bias) and three domains that may increase it (i.e., large effect, dose-response gradient, and plausibility of confounding). The following rating system was used:

- *Very Confident:* Rating up two levels (↑2 levels)
- *Confident:* Rating up one level (↑1 level)
- *No Concern: *No rating down (0 score)
- *Some Concerns:* Rating down by 0.5 level (↓0.5 level)
- *Serious Concerns:* Rating down by one level (↓1 level)
- *Very Serious Concerns:* Rating down by two levels (↓2 levels)

Based on these criteria, the GRADE system classifies evidence into four levels of certainty:

1. *Very Low Certainty:* The evidence is insufficient to draw any reliable conclusions.
2. *Low Certainty:* The evidence is limited, and there is a high risk of bias or inconsistency.
3. *Moderate Certainty:* The evidence is moderate, with some limitations in the quality or consistency of the findings.
4. *High Certainty:* The evidence is strong, with minimal risk of bias or inconsistency.

A cumulative meta-analysis was performed to assess confidence in the overall effect estimate. This method involves a sequential series of meta-analyses, starting with the earliest study and progressively adding subsequent studies. By tracking changes in the overall effect size with each addition, cumulative meta-analysis provides insight into how the evidence evolves over time. In addition, combining results from multiple studies increases the sample size and yields more precise estimates of the true effect size, as reflected by narrower CIs. This approach enhances confidence in the conclusions drawn regarding the research question.

## Results

###  Study Selection and Characteristics 

 Initially, a total of 15,586 studies were identified, consisting of 11,910 studies obtained through electronic database searches and an additional 3,676 studies identified by screening the reference lists of the included studies. After removing duplicate studies and excluding those not meeting the eligibility criteria, 141 studies were included in this systematic review (Figure 1). These studies involved a total of 1,045,983 participants. Among the included studies, there were 11 cohort studies and 130 case-control studies (Supplementary Table 1).

**Figure 1:**
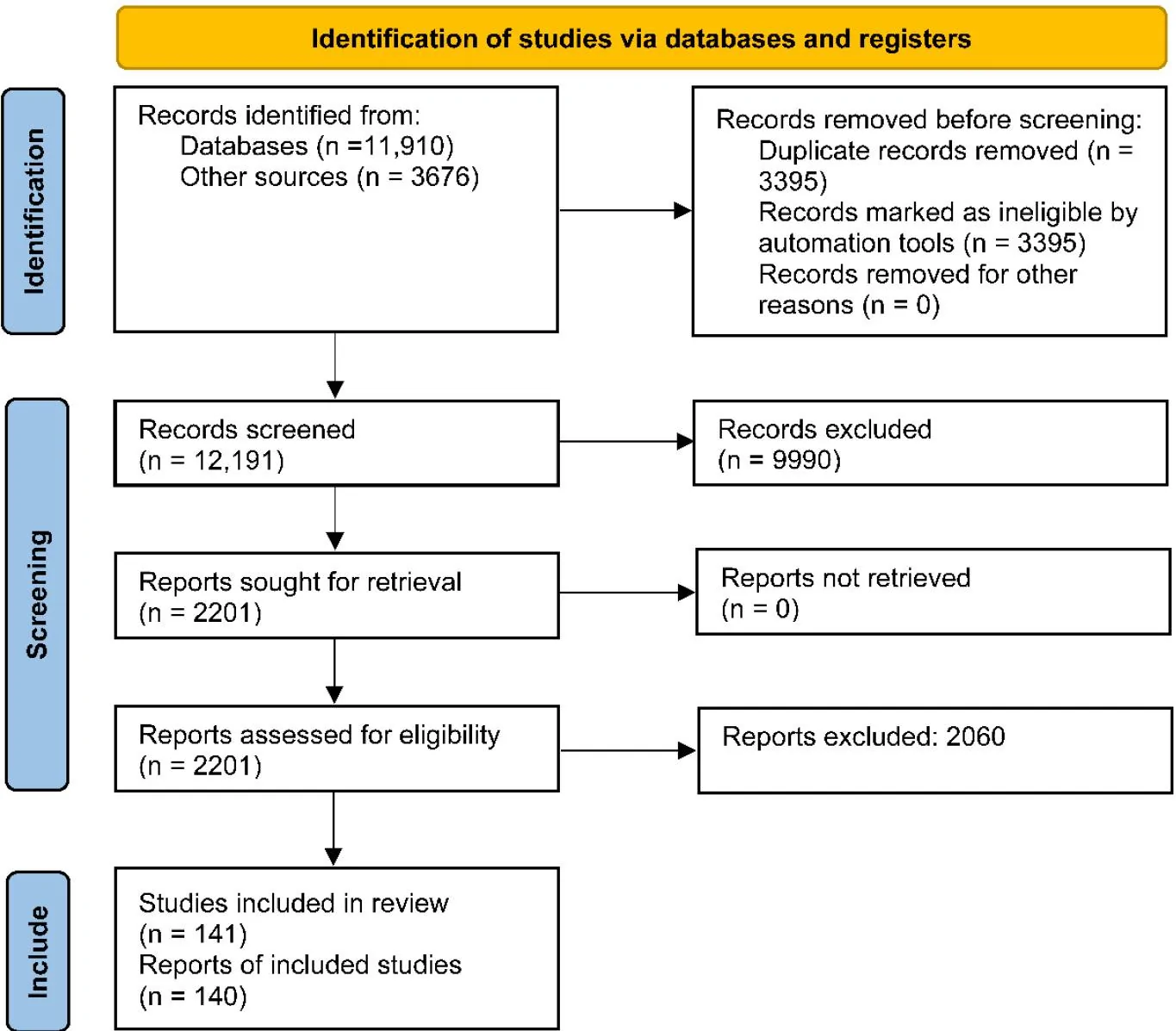
Flow Diagram of the Study Selection Process in the Systematic Review

###  Results of Syntheses

 From the analysis of 10 cohort studies (Figure 2), the overall RR for age at first pregnancy ≥ 20 years compared to < 20 years was 1.18 (95% CI: 1.09–1.28). The overall effect measure demonstrated that women who have their first pregnancy at the age of 20 or older have an approximately 18% higher probability of developing BC (*P* < 0.0001). There was moderate between-study heterogeneity, as indicated by an I^2^ value of 53%.

**Figure 2:**
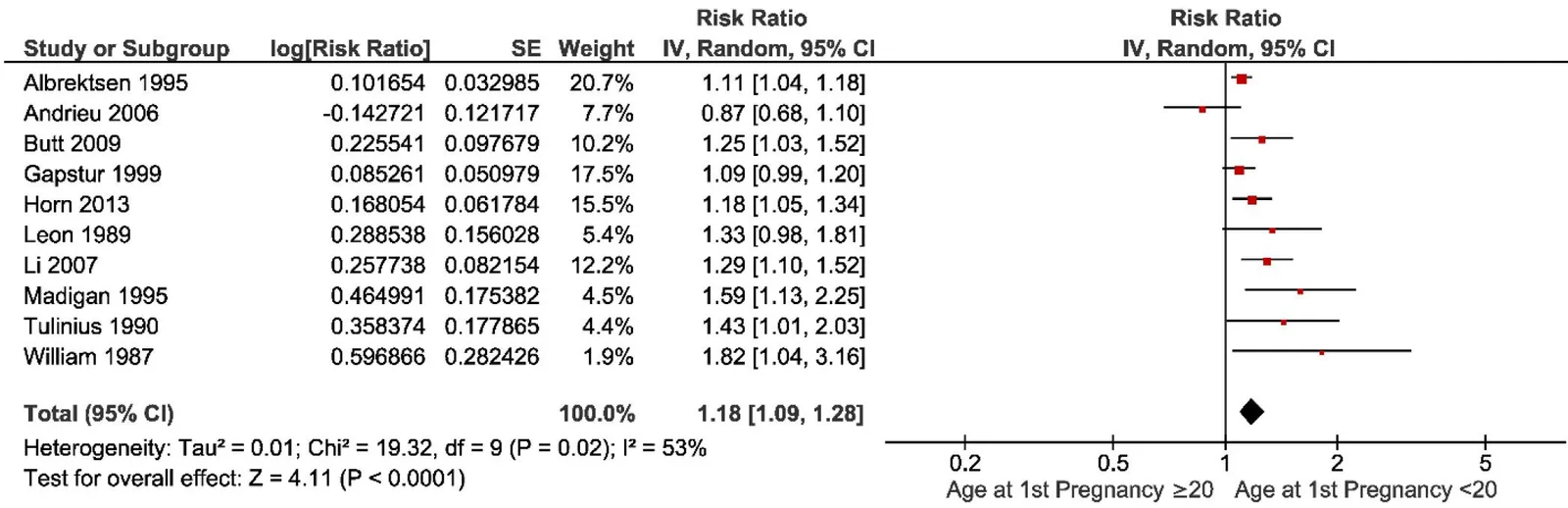
Forest Plot of Cohort Studies Evaluating Breast Cancer Risk Associated With Age at First Pregnancy ≥ 20 Years Compared to < 20 Years, With Age < 20 as the Reference Group
*Note*. SE: Standard error; CI: Confidence interval.

 One cohort study^28^ was identified as eligible based on our criteria; however, it was not included in the meta-analysis due to the significant heterogeneity it introduced to the overall effect estimate. This particular study involved 29,508 Swedish women aged 25–65 years. The study reported an RR of BC 1.00 (95% CI: 0.98–1.03) for age at first pregnancy ≥ 20 years compared to < 20 years.

 According to the analysis of 130 case-control studies, the overall OR for age at first pregnancy ≥ 20 years compared to < 20 years was 1.31 (95% CI: 1.26–1.37). The overall effect measure revealed that women who have their first pregnancy at the age of 20 or older have nearly a 31% higher probability of developing BC (*P* < 0.00001). High between-study heterogeneity was observed, with an I^2^ value of 81% (Supplementary Figure 1).

###  Sensitivity Analysis

 To address the high between-study heterogeneity found in the case-control studies, a sensitivity analysis was performed using a sequential technique. This involved systematically excluding one study at a time to assess the impact on homogeneity. Through this process, it was possible to achieve homogeneity with an I^2^ value of 45% when the number of studies was reduced to 111.

 Following the sensitivity analysis, a slight reduction was observed in the magnitude of the association (Supplementary Figure 2). The OR for age at first pregnancy ≥ 20 years versus < 20 years was calculated to be 1.21 (95% CI: 1.17–1.24), implying that if the results of the case-control studies were homogeneous, having the first pregnancy at the age of 20 or older would increase the probability of BC by approximately 21% (*P* < 0.001).

###  Stratified Meta-Analysis

Figure 3 displays the outcomes of the stratified meta-analysis investigating the associations between age at first pregnancy and BC, focusing on cohort studies. The findings confirmed a positive and statistically significant trend, demonstrating an increased incidence of BC with higher age at first pregnancy. Within specific age groups, between-study heterogeneity was low (I^2^ < 50%). However, overall heterogeneity was moderate (I^2^ = 62%), indicating variations in the risk of BC across different age groups.

**Figure 3:**
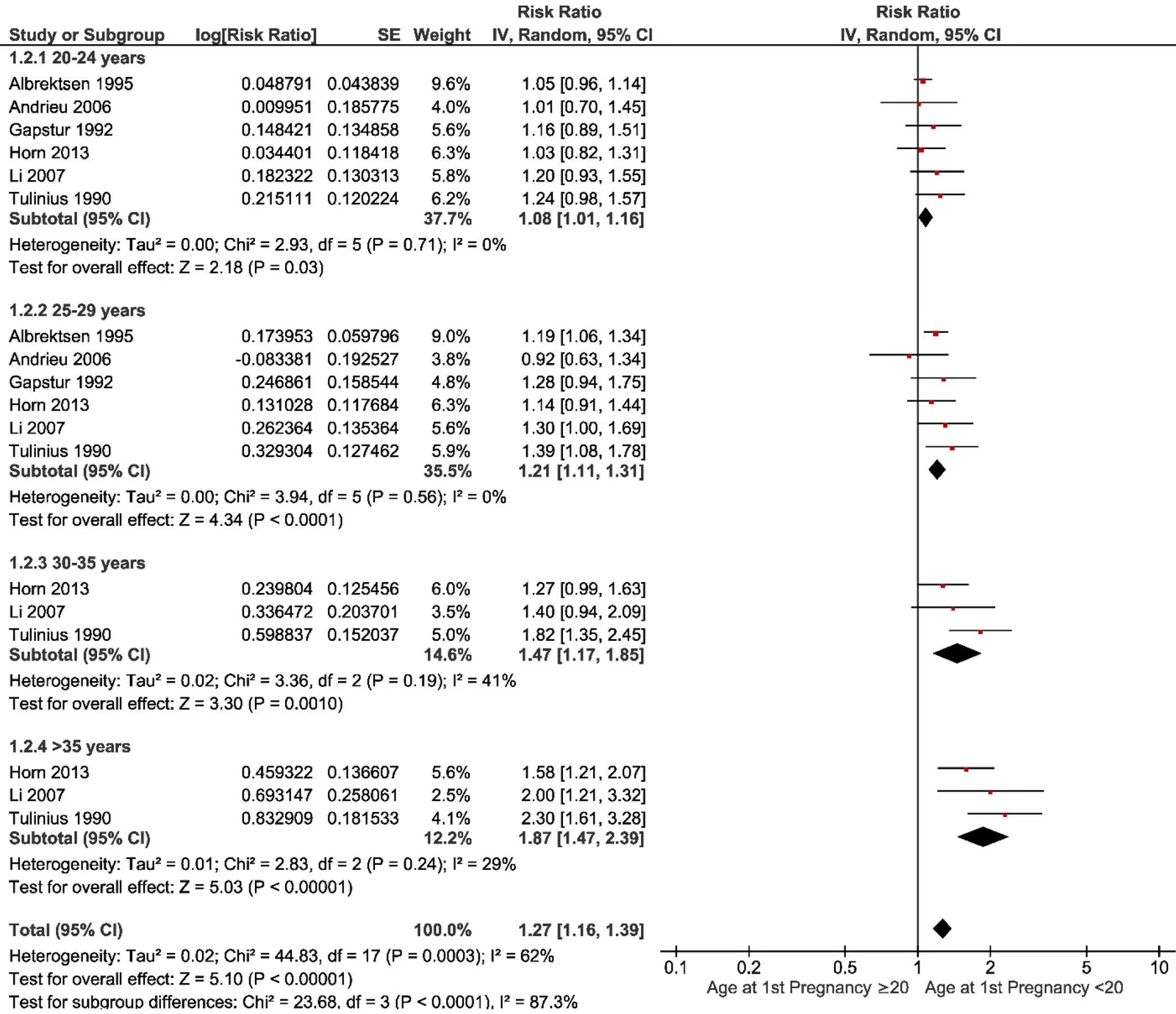
Forest Plot of Cohort Studies Investigating the Association Between Age at First Pregnancy and Breast Cancer Risk, Stratified by Age at First Pregnancy, With Age < 20 as the Reference Group
*Note*. SE: Standard error; CI: Confidence interval.

 Due to the large number of studies across subgroups, the forest plot illustrating the stratified meta-analysis investigating the associations between age at first pregnancy and BC based on case-control studies was not included in this text. Including all the studies would have compromised the quality of the figure, making it difficult to read. However, Figure 4 displays the results of the stratified meta-analysis investigating the associations between age at first pregnancy and BC, incorporating both cohort and case-control studies. The findings from cohort studies represented a consistent upward trend, implying an increased incidence of BC with higher age at first pregnancy. However, the results of case-control studies revealed a trend that was not uniformly steady. Specifically, the risk of BC in the age group of 25–29 years was higher compared to the age group of 30–34 years.

**Figure 4:**
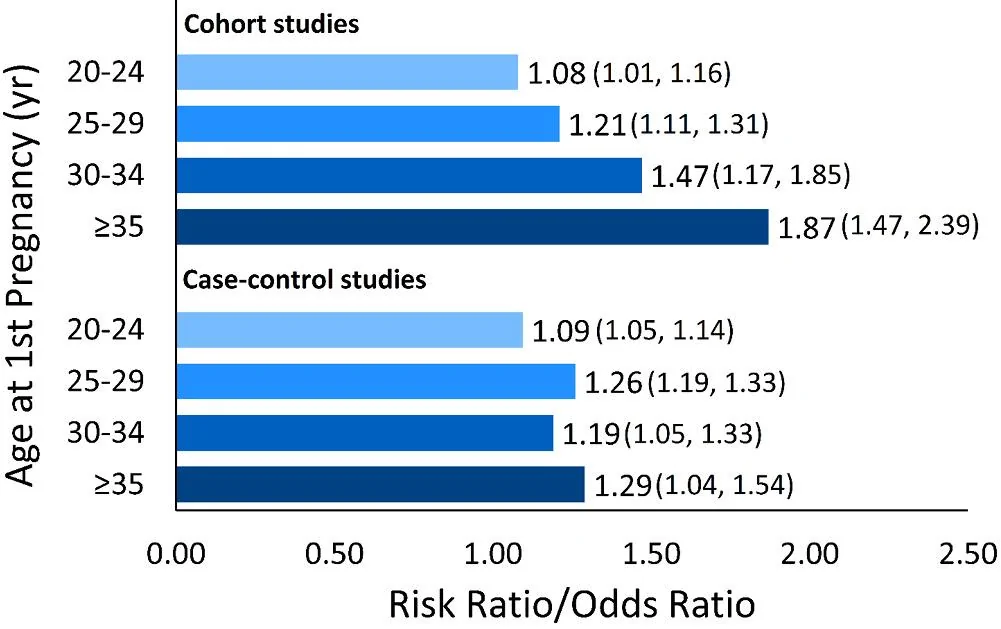
An Overview of Stratified Associations (95% CI) Between Age at First Pregnancy and Breast Cancer Risk in Cohort and Case-Control Studies
*Note*. CI: Confidence interval

###  Dose-Response Meta-Analysis

 The results of the dose-response meta-analysis indicated a significant association between age at first pregnancy and BC risk (Table 1). For cohort studies, the RR of 1.083 showed that each 5-year increase in age at first pregnancy is associated with an 8.3% increase in the risk of developing BC, with a *P*-value of 0.048 highlighting statistical significance. Similarly, for case-control studies, the OR of 1.125 confirmed a 12.5% increase in the odds of BC for each 5-year increment in age at first pregnancy, with a highly significant *P*-value of less than 0.001. The 95% CIs for both studies designs further support the robustness of these findings, suggesting that delaying the age of first pregnancy may be associated with an increased risk of BC.

**Table 1:** Dose-Response Meta-Analysis of the Relationship Between Age at First Pregnancy and Breast Cancer Risk, Expressed as RRs for Cohort Studies and ORs for Case-Control Studies, per 5-Year Increase in Age, Using Age < 20 as the Reference Group

**Study Design**	**Number of Studies**	**RR/OR**	**SE**	**z**	***P*** **-Value**	**95% CI**	
Cohort	6	1.083	0.044	1.98	0.048	1.001	1.173
Case-control	86	1.125	0.010	13.13	0.000	1.106	1.145

*Note*. RR: Risk ratio; OR: Odds ratio; CI: Confidence interval; SE: Standard error.

Figure 5 depicts the association between age at first pregnancy and BC risk (a clear increasing linear trend). In Figure 5a, for cohort studies, the points reflect the RRs that consistently rise with each subsequent increase in age at first pregnancy, reinforcing an 8.3% increase in risk for every 5-year increment. Similarly, Figure 5b for case-control studies shows an upward trend in ORs, highlighting a 12.5% increase in odds for each additional 5 years. These trends underscore the robust relationship between delayed first pregnancy and heightened BC risk across different study designs, further supporting the findings of the meta-analysis.

**Figure 5:**
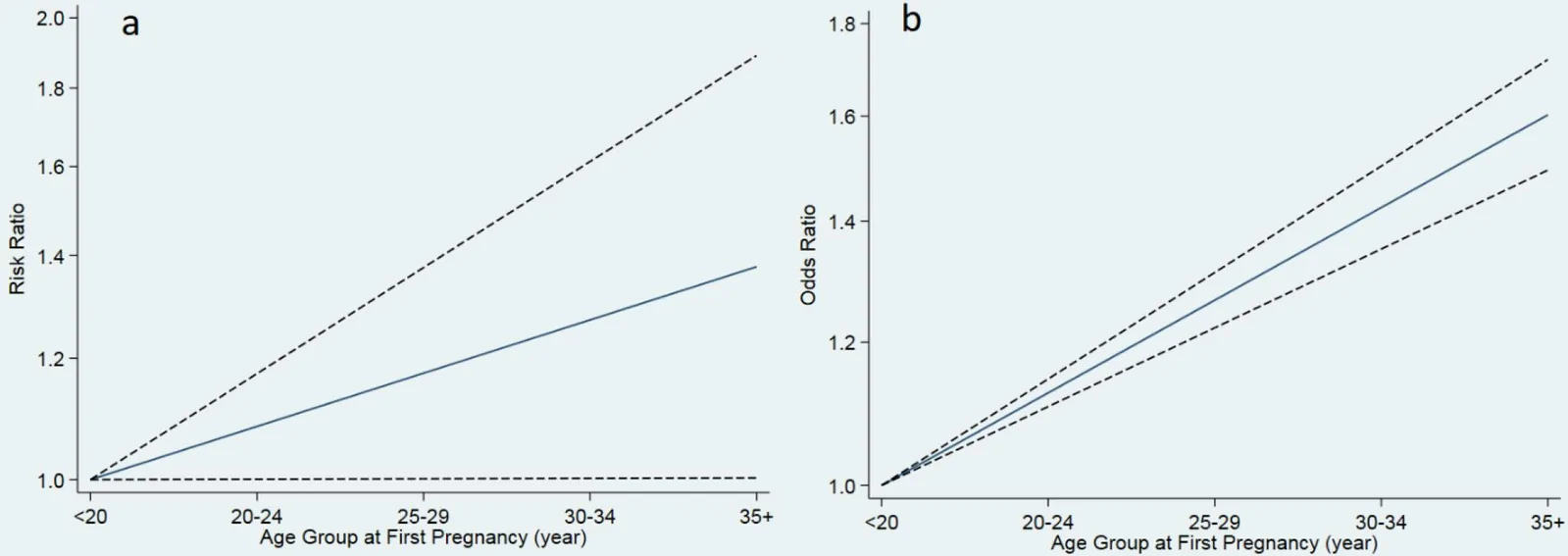
Dose-Response Meta-Analysis of the Association Between Age at First Pregnancy and Breast Cancer Risk per 5-Year Increment: **(a)** Cohort Studies and **(b)** Case-Control Studies

###  Publication Biases

 In the cohort studies, the Begg’s test showed no statistically significant evidence of publication bias (*P* = 0.533), whereas the Egger’s test indicated a statistically significant result (*P* < 0.001). In the case-control studies, both the Begg’s test (*P* < 0.001) and the Egger’s test (*P* < 0.001) demonstrated significant publication bias in the analysis of the association between age at first pregnancy and BC.

 To address this potential bias, a “Trim and Fill” analysis was performed for both the cohort and case-control studies to adjust the overall estimates of the meta-analysis. This analysis identified 3 potentially missing studies for the cohort studies (Figure 6) and 22 potentially missing studies for the case-control studies (Figure 7).

**Figure 6:**
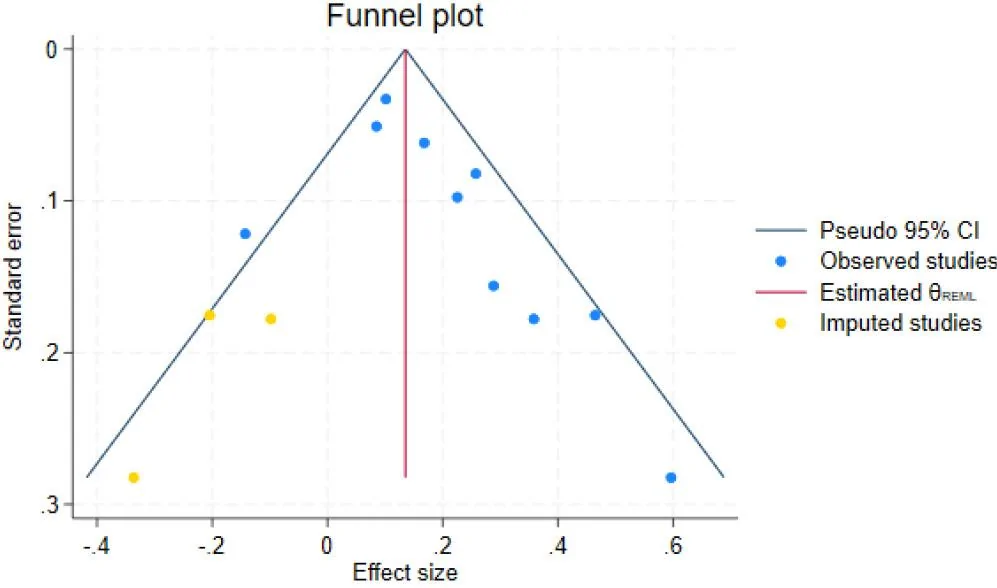
Funnel Plot of Cohort Studies Examining the Relationship Between Age at First Pregnancy and Breast Cancer Risk, Highlighting Potentially Missing Studies With Squares, Based on Trill and Fill Analysis
*Note*. CI: Confidence interval

**Figure 7:**
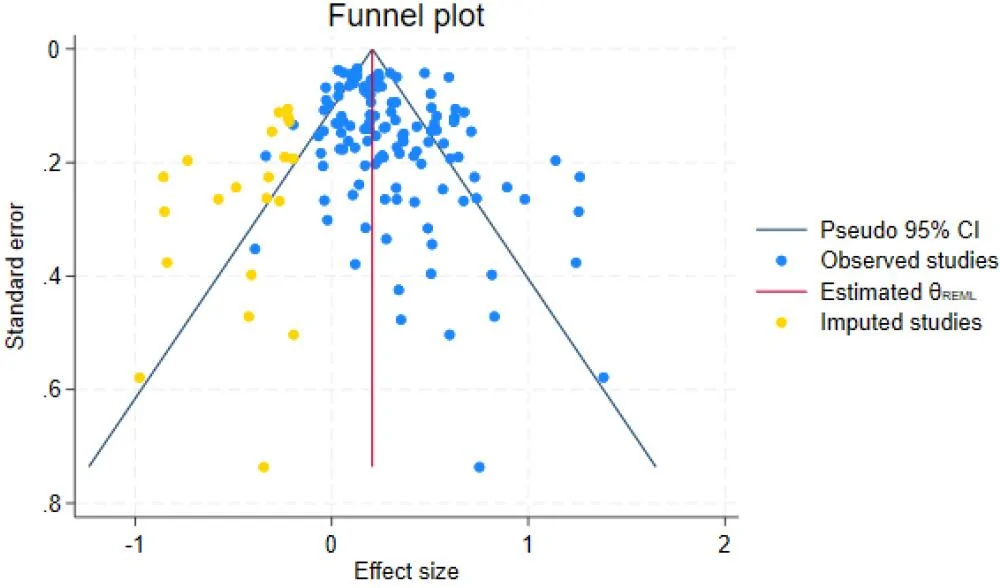
Funnel Plot of Case-Control Studies Investigating the Relationship Between Age at First Pregnancy and Breast Cancer Risk, Indicating Potentially Missing Studies With Squares, Based on Trill and Fill Analysis
*Note*. CI: Confidence interval

Table 2 presents a comparison of the observed overall effect estimates with the adjusted (observed + imputed) estimates. Compared to the observed estimates, the adjusted overall effect estimates were slightly attenuated in both the cohort and case-control studies. However, the differences were not statistically significant.

**Table 2:** Sensitivity Analysis Using “Trim and Fill” to Address Publication Bias

**Study Design**	**Publication Bias**	**Number of Studies**	**RR/OR (95% CI)**
Cohort	Observed	10	1.18 (1.09, 1.28)
	Observed + Imputed	13	1.14 (1.04, 1.25)
Case-control	Observed	130	1.31 (1.26, 1.37)
	Observed + Imputed	152	1.23 (1.17, 1.29)

*Note*. RR: Risk ratio; OR: Odds ratio; CI: Confidence interval. This analysis adjusts the overall effect estimates by accounting for potential missing or unpublished studies. The table compares the observed results with the adjusted (observed + imputed) results, incorporating potentially missing or unpublished studies.

###  Meta-Regression

 A multivariate meta-regression analysis was conducted to explore the potential sources of heterogeneity across the case-control studies. This analysis took into account various factors, including study settings based on World Health Organization regions, publication year, sample size, and the adjusted or unadjusted effect size. Nonetheless, the results of the meta-regression analysis indicated that none of the examined factors could significantly influence the observed heterogeneity. Although the publication year showed statistical significance (*P* = 0.045), it did not have a significant impact on the overall observed heterogeneity. In other words, no strong and significant factors could explain the variation observed across the case-control studies (Table 3). Nevertheless, homogeneity in the results was achieved when a stratified meta-analysis was performed based on different age groups, suggesting that the variation in the association between age at first pregnancy and BC across different age groups may contribute to the overall heterogeneity observed in the cohort and case-control studies.

**Table 3:** Meta-Regression Results Identifying the Potential Sources of Heterogeneity in Case-Control Studies

**Variables**	**Coefficient**	**SE**	**t**	***P*** **-Value**	**95% CI**	
WHO regions	-0.02	0.02	-1.19	0.237	-0.06	0.01
Publication year (per 10 years)	0.05	0.02	2.03	0.045	0.00	0.10
Sample size	0.05	0.03	1.70	0.091	-0.01	0.10
Adjust/unadjusted effect size	0.07	0.04	1.68	0.095	-0.01	0.16
Constant	-0.51	0.09	-5.66	0.000	-0.69	-0.33

*Note*. WHO: World Health Organization; SE: Standard error; CI: Confidence interval. This analysis explores possible sources of heterogeneity across studies by including variables that may contribute to variations in effect sizes.

###  Risk of Bias in Studies 

 The NOS was utilized to evaluate the methodological quality of the studies included in the analysis. According to this scale, out of the 140 studies included in the meta-analysis, 72 were classified as high-quality, while 68 were considered low-quality.

Table 4 summarizes the effect sizes of the association under investigation, stratified by study design, study quality, and adjustment for confounding factors. Among the cohort studies, the pooled relative risk (RR) was 1.18, indicating a modest but statistically significant association. Higher-quality cohort studies demonstrated a slightly stronger association (RR = 1.20) than lower-quality studies (RR = 1.14), suggesting that more rigorous study designs yield more reliable estimates of the effect.

**Table 4:** Sensitivity Analysis of Effect Size Based on Study Quality (NOS Scale) and Confounding Adjustments

	**Cohort Studies**		**Case-Control Studies**	
**Variables**	**Number of Studies**	**RR (95% CI)**	**Number of Studies**	**OR (95% CI)**
Quality of the studies				
Total	10	1.18 (1.09, 1.28)	130	1.30 (1.26, 1.35)
High Low	8	1.20 (1.09, 1.34)	64	1.25 (1.19, 1.31)
	2	1.14 (0.96, 1.35)	66	1.37 (1.30, 1.45)
Confounding adjustments				
Total	10	1.18 (1.09, 1.28)	130	1.30 (1.26, 1.35)
Adjusted effect sizes Crude effect sizes	8	1.16 (1.07, 1.26)	68	1.27 (1.20, 1.33)
	2	1.43 (1.10, 1.87)	62	1.35 (1.28, 1.43)

*Note*. RR: Risk ratio; OR: Odds ratio; CI: Confidence interval; NOS: Newcastle-Ottawa Scale. This analysis compared effect sizes by study quality, as rated by the NOS scale, and by whether included studies reported crude or adjusted effect sizes.

 In contrast, case-control studies showed a stronger overall association, with a pooled OR of 1.30. However, the association was more pronounced in low-quality case-control studies (OR = 1.37) compared with high-quality studies (OR = 1.25), representing the possibility of bias or residual confounding influencing results from less rigorous studies.

 Stratification by confounding adjustment revealed that adjusted effect estimates were generally lower than crude estimates in both study designs. In cohort studies, adjusted analyses yielded an RR of 1.16 compared with a higher crude RR of 1.43, while in case-control studies, adjusted ORs (1.27) were slightly lower than crude ORs (1.35). These findings underline the importance of controlling for confounding variables when estimating the true magnitude of the association.

###  Certainty of Evidence

 There were very serious concerns regarding the overall “risk of bias”. Some concerns were noted about “inconsistency” across studies. Conversely, there were no concerns about “indirectness” in addressing the review question, nor about “imprecision”. However, serious concerns were found regarding “publication bias”. There was moderate confidence in the “effect size”. Moreover, a dose-response gradient was observed across subgroups, and confounding factors were unlikely to fully explain the findings. Based on these assessments, we are moderately confident that there is an increasing trend in the incidence of BC with higher age at first pregnancy.

 To assess confidence in the overall effect estimate, a cumulative meta-analysis was conducted for both cohort and case-control studies, the results of which are shown in Figure 8 and Supplementary Figure 3, respectively.

**Figure 8:**
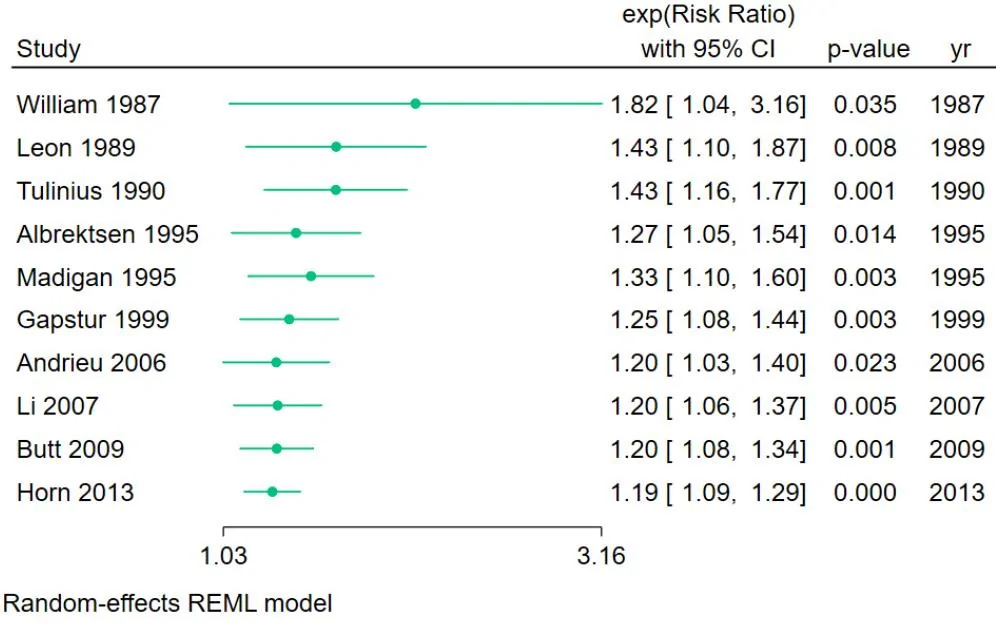
Cumulative Meta-Analysis of Cohort Studies Assessing Age at First Pregnancy and Breast Cancer Risk, Showing Sequential and Chronological Meta-Analyses With the Progressive Addition of Studies to Evaluate Robustness of the Overall Association
*Note*. CI: Confidence interval; REML: Restricted maximum likelihood

 For cohort studies, the forest plot indicated a relatively precise estimate of the true effect size, suggesting that the cumulative evidence from cohort studies provides a relatively robust and reliable estimate of the association between age at first pregnancy and BC, although it may not be perfect.

 On the other hand, the forest plot of the case-control studies revealed narrower CIs and a more precise estimate of the true effect size, indicating that the cumulative evidence from case-control studies also presents a strong and reliable estimate of the association between age at first pregnancy and BC.

## Discussion

 This systematic review and meta-analysis investigated the association between age at first pregnancy and the risk of BC. Based on the results, both cohort and case-control studies demonstrated a positive association between age at first pregnancy and BC risk. In addition, the dose-response meta-analyses for types of studies showed a consistent upward trend, implying an increased incidence of BC with higher age at first pregnancy.

 The exact mechanisms underlying the association between higher age at first pregnancy and increased BC risk are not yet fully understood. However, several hypotheses have been proposed to explain this relationship. One possible explanation is related to breast cell proliferation and differentiation that occur during pregnancy and lactation. Late pregnancy may increase the risk of BC due to the biological differences in breast tissues. Women who give birth at a young age, especially before 24 years old, have a lower lifetime risk of BC. Additional pregnancies further decrease the risk. The breast tissue changes during pregnancy and lactation, with the most developed stage being Lobules type 4. After menopause, the breast regresses and contains only Lobules type 1. Nulliparous women have a higher risk of BC compared to parous women, demonstrating that Lobules type 1 may be biologically different or more susceptible to carcinogenesis in nulliparous women. It is hypothesized that Lobules type 1 in nulliparous women retains a high concentration of epithelial cells that are targets for carcinogens, while, in early parous women, it is composed of epithelial cells that are resistant to transformation and cancer development. These differences may be due to changes in specific stem cells within the breast.^29,30^ On the other hand, pregnancy and lactation induce changes in the structure and function of the mammary glands. Early pregnancies promote complete differentiation and maturation of breast tissues, making the breasts less susceptible to carcinogenic events. In contrast, delayed or nulliparous pregnancies may result in the incomplete maturation of the mammary glands, leaving them more vulnerable to genetic and environmental factors that can contribute to BC development.^31^ Furthermore, age at first pregnancy is commonly associated with various lifestyle and reproductive factors that may independently influence breast cancer risk. For instance, women who delay pregnancy may have a higher likelihood of engaging in behaviors such as alcohol consumption, sedentary lifestyle, or use of hormonal contraceptives, which are themselves associated with an increased risk of BC.^32,33^ However, further research is needed to fully understand these mechanisms.

 To ensure a comprehensive assessment of the association between age at first pregnancy and BC risk, we carefully considered the appropriate age range for our control group. While the World Health Organization generally defines the childbearing age as 15–49 years old, studies focusing on early pregnancy frequently include women aged 10–19 to capture the full spectrum of adolescent pregnancies. This age range has been identified as a high-risk group in previous research, with the increased risks of maternal and neonatal health complications.^15^ By including women aged 10–19 in our control group, it was possible to establish a robust comparison and more accurately evaluate the potential impact of age at first pregnancy on BC risk. This approach allowed us to examine the association across a wider range of ages, providing a more nuanced understanding of the relationship between these two factors.

 While this meta-analysis revealed a positive link between the age at first pregnancy and BC, it is important to remember that BC risk is not determined by a single factor alone. It is typically the result of a complex interaction between multiple risk and protective factors.^32,33^ These factors cannot be separated into distinct components and should be considered as a whole. Risk factors increase the likelihood of BC, while protective factors decrease it. In this context, if the risk and protective factors are balanced, or if the protective factors outweigh the risk factors, BC is less likely to occur.^34,35^ Therefore, other influential factors should also be considered when assessing the role of age at first pregnancy in BC development.

 Unlike cohort studies, case-control studies showed high levels of heterogeneity in this meta-analysis. The meta-regression results indicated that study characteristics (e.g., study settings, publication year, sample size, and method of analysis) were not responsible for the observed heterogeneity. However, a stratified meta-analysis conducted based on different age groups helped achieve homogeneity in the results. This implies that variations in the association between age at first pregnancy and BC in different age groups are probably contributed to the overall heterogeneity in the case-control and cohort studies.

 The study acknowledges the presence of significant publication bias in the meta-analysis, which could affect the validity of the overall findings. A statistical technique called “Trim and Fill” analysis was conducted to address this bias. This analysis helps adjust the overall effect estimates by accounting for potential missing or unpublished studies. The adjusted overall effect estimates were found to be slightly weaker but not statistically different from the original estimates. This suggests that the positive association between age at first pregnancy and BC risk remains even after accounting for publication bias. However, it is important to interpret these findings cautiously, as the presence of publication bias introduces some uncertainty to the results.

 The results of the stratified meta-analysis from cohort studies consistently demonstrated an upward trend. Contrarily, the findings from case-control studies indicated a less consistent pattern. This discrepancy could be attributed to the potential misclassification of age at first pregnancy in case-control studies.

 This review had several limitations and potential biases that should be taken into account when interpreting the findings. First, there was evidence of significant publication bias in the association between age at first pregnancy and BC, which could impact the accuracy of the overall estimates. In addition, the studies included in the analysis had varying levels of quality, and certain studies may have had biases or limitations that could have influenced the reliability of their results. Moreover, this meta-analysis relied on aggregated data from published studies rather than individual patient data, thereby limiting the ability to adjust for potential confounding factors at the individual level and perform more detailed subgroup analyses. Furthermore, the potential misclassification of age at first pregnancy in the case-control studies could introduce bias and affect the accuracy of the overall effect estimates. Finally, the meta-analysis of observational studies cannot establish causality between age at first pregnancy and BC risk, but only an association. Therefore, it is crucial to bear in mind these limitations when interpreting the results of the meta-analysis and when applying the findings to clinical practice or making public health recommendations.

 Although the association between age at first pregnancy and BC risk observed in this study is noteworthy, it should be interpreted with caution due to the limitations of the included studies and the moderate strength of the evidence. Likewise, these findings may contribute to risk stratification efforts but should not be used in isolation to guide clinical decision-making or public health recommendations.

 Public awareness initiatives may consider including information about reproductive factors as part of broader BC education; however, reproductive choices are complex and influenced by social, economic, and personal considerations; thus, they should not be directed solely based on cancer risk. Similarly, while age at first pregnancy could be explored as one component of comprehensive risk assessment models, current evidence does not justify changes to existing BC screening guidelines solely on this basis.

 Accordingly, further well-designed prospective cohort studies with careful control of confounding variables are needed to strengthen the evidence base and clarify the potential clinical relevance of this association.

**Highlights**

- First pregnancy ≥20 years increased breast cancer risk.
- Cohort studies demonstrated 18% higher risk (risk ratio: 1.18).
- Case-control studies showed 31% higher odds (odds ratio: 1.31).
- Breast cancer risk increased 8–12% per 5-year delay in the first pregnancy.
- Dose-response analysis confirmed a significant linear trend.

## Conclusion

 This meta-analysis provided moderate evidence supporting a positive association between age at first pregnancy and the risk of BC. It was revealed that women who have their first pregnancy at the age of 20 or older are at a higher risk of developing BC compared to those with a first pregnancy before the age of 20. While the findings highlight the importance of considering age at first pregnancy as a potential risk factor in BC prevention and screening strategies, it is essential to acknowledge the limitations of the evidence. Heterogeneity among studies and potential publication biases may have influenced the observed association. To further clarify the underlying mechanisms and explore variations in the association across different age groups, population-based prospective cohort studies with robust methodological designs are necessary.

## Acknowledgments

 These results were derived from an MSc thesis in epidemiology. The authors gratefully acknowledge the Vice-Chancellor for Research and Technology of Hamadan University of Medical Sciences for approving and supporting this study (No. 14030204759; IR.UMSHA.REC.1403.049).

## Artificial Intelligence Use Disclosure

 The authors used artificial intelligence tools solely for language editing, grammar improvement, and assistance with abstract preparation rather than for data extraction, data analysis, figure generation, or interpretation of the results.

## Competing Interests

 The authors declare that they have no conflict of interests related to this study.

## Ethical Approval

 This study was a systematic review and meta-analysis based exclusively on data from previously published studies. No ethical approval or informed consent was required since no new data were collected from human participants or animals.

## Funding

 This study received approval from the Vice-Chancellor of Research and Technology at Hamadan University of Medical Sciences (14030204759). No external funding was obtained for this research.

## Supplementary Files

Supplementary file 1 contains Table S1 and Figures S1 and S2.
