# Supplementary material for: A Systematic Review and Dose-Response Meta-Analysis of the Association Between the Age of First Pregnancy and Breast Cancer Risk
Source: J Res Health Sci. 2026 Aug 15;26(3):e00685. doi: 10.34172/jrhs.13923 (PMC13698876; doi:10.34172/jrhs.13923)

Supplementary Table 1: Characteristics of included studies [1-141], sorted by authors' names

ID	Autor	Country	Age mean/range (yr)	Study	adj	Sample size	NOS score	Quality
1	Adami 1978	Sweden	63.00	Case-control	Adjusted	358	★★★★★★★	High
2	Adami 1980	Sweden	63.50	Case-control	Adjusted	2,002	★★★★★	Low
3	Adami 1990	Sweden & Norway	Unspecified	Case-control	Adjusted	949	★★★★★★★★	High
4	Albrektsen 1995	Norway	20-56	Cohort	Adjusted	82,457	★★★★★★★	High
5	Andrieu 2006	Europe	46.70	Cohort	Adjusted	1,601	★★★★★★★	High
6	Austin 1979	USA	49.93	Case-control	Unadjusted	444	★★★★★★	Low
7	Babita 2012	India	49.76	Case-control	Adjusted	256	★★★★★★	Low
8	Bain 1981	USA	30-55	Case-control	Adjusted	12,749	★★★★★★★	High
9	Barbon 1996	Italy	Unspecified	Case-control	Unadjusted	5,157	★★★★★★	Low
10	Baron 1984	UK	Unspecified	Case-control	Adjusted	2,352	★★★★★★★★	High
11	Bernstein 1994	USA	<40	Case-control	Unadjusted	1,090	★★★★★★	Low
12	Bouchardy 1990	France	58.10	Case-control	Unadjusted	2,960	★★★★★★	Low
13	Brignone 1987	Italy	54.29	Case-control	Unadjusted	1,706	★★★★★★	Low
14	Brinton 1978	USA	>35	Case-control	Adjusted	1965	★★★★★★	Low
15	Brinton 1982	USA	Unspecified	Case-control	Unadjusted	1,821	★★★★★	Low
16	Brinton 1983	USA	Unspecified	Case-control	Unadjusted	2,612	★★★★★★★	High
17	Brinton 1986	USA	Unspecified	Case-control	Unadjusted	3,477	★★★★★★	Low
18	Brinton 1995	USA	20-54	Case-control	Adjusted	3,153	★★★★★★★	High
19	Bruzzi 1985	USA	Unspecified	Case-control	Unadjusted	1,897	★★★★★★	Low
20	Bruzzi 1988	Italy	<75	Case-control	Adjusted	1,143	★★★★★★★★	High
21	Buchanan 1985	USA	30-40	Case-control	Unadjusted	1,109	★★★★★★	Low
22	Butt 2009	Sweden	57.3	Cohort	Adjusted	17,035	★★★★★★★	High
23	Butt 2012	Sweden	Unspecified	Case-control	Adjusted	2,176	★★★★★★★★	High
24	Byers 1985	USA	40-84	Case-control	Unadjusted	1,818	★★★★★★★	High
25	Byrne 1991	USA	>35	Case-control	Unadjusted	6,088	★★★★★★★	High
26	Chen 2002	USA	50-74	Case-control	Adjusted	1,397	★★★★★★★	High
27	Chie 2000	USA	20-79	Case-control	Adjusted	22,162	★★★★★★★★	High
28	Choi 1978	Canada	35-74	Case-control	Adjusted	800	★★★★★★★	High
29	CHS study	USA	20-54	Case-control	Unadjusted	9,387	★★★★★★	Low
30	Clave 1991	France	44.17	Case-control	Unadjusted	1,006	★★★★★★	Low
31	Clavel-Chapelon 1994	France	22-58	Case-control	Adjusted	1,037	★★★★★★★★	High
32	Coogan 1999	South Africa	≤50	Case-control	Unadjusted	1,918	★★★★★★	Low
33	Cooper 1989	Australia	20-74	Case-control	Adjusted	902	★★★★★★★	High
34	Craig 1974	USA	Unspecified	Case-control	Adjusted	394	★★★★★★★	High

ID	Autor	Country	Age mean/range (yr)	Study	adj	Sample size	NOS score	Quality
35	Daling 2002	USA	<65	Case-control	Unadjusted	3,702	★★★★★★	Low
36	Enger 1997	USA	<40	Case-control	Adjusted	904	★★★★★★★★★	High
37	Enger 1998	USA	55-64	Case-control	Adjusted	1,947	★★★★★★★★	High
38	Ewertz 1988	Denmark	<70	Case-control	Adjusted	2,822	★★★★★★★★	High
39	Ewertz 1992	Denmark	<59	Case-control	Unadjusted	2,050	★★★★★★	Low
40	Freudenheim 1994	USA	40-85	Case-control	Unadjusted	1,130	★★★★★★	Low
41	Furberg 1999	USA	20-74	Case-control	Unadjusted	1,493	★★★★★	Low
42	Gajalakshmi 1991	India	Unspecified	Case-control	Adjusted	1,062	★★★★★★★★	High
43	Gao 2000	China	47.60	Case-control	Adjusted	3,015	★★★★★	Low
44	Gapstur 1992	USA	55-69	Cohort	Adjusted	41,837	★★★★★★★★	High
45	Gapstur 1999	USA	55-69	Cohort	Adjusted	37,105	★★★★★★	Low
46	Gilliland 1998	Mexico	30-74	Case-control	Adjusted	1,555	★★★★★★★★	High
47	Haile 1996	USA	41.00	Case-control	Adjusted	180	★★★★★	Low
48	Harris 1982	USA	35-54	Case-control	Unadjusted	578	★★★★★★	Low
49	Helmrich 1983	USA, Canada, Israel	52.00	Case-control	Adjusted	4,412	★★★★★★★★★	High
50	Henderson 1974	USA	<64	Case-control	Adjusted	616	★★★★★★★★★	High
51	Horn 2013	Norway	28-73	Cohort	Adjusted	58,426	★★★★★★★★★	High
52	Hsieh 1996	7 nations	>35	Case-control	Adjusted	15,460	★★★★★★★★★	High
53	Hunt 1979	USA	63.50	Case-control	Unadjusted	1,173	★★★★★★	Low
54	Innes 2000	USA	14-37	Case-control	Unadjusted	3,388	★★★★★★★★★	High
55	Jick 1980a	USA	45-64	Case-control	Unadjusted	261	★★★★★★	Low
56	Jick 1980b	USA	31-55	Case-control	Unadjusted	341	★★★★★	Low
57	Jick 1989	USA	<43	Case-control	Adjusted	301	★★★★★★★★	High
58	Kalache 1993	Brazil	Unspecified	Case-control	Unadjusted	1,018	★★★★★	Low
59	Khalis 2018	Morocco	48.60	Case-control	Adjusted	474	★★★★★★★★★	High
60	Kinlen 2014	7 nations	>20	Case-control	Unadjusted	17,022	★★	Low
61	Kinlen 2020	UK	15-64	Case-control	Unadjusted	20,483	★★★★★★	Low
62	Kotsopoulos 2007	11 countries	47.15	Case-control	Adjusted	6,366	★★★★★★★★★	High
63	Kuru 2002	Turkey	47.75	Case-control	Adjusted	1,114	★★★★★★★★★	Low
64	La vecchia 1987	Italy	25-74	Case-control	Adjusted	2,389	★★★★★★★★★	High
65	La vecchia 1989	Italy	21-59	Case-control	Unadjusted	2,868	★★★★★	Low
66	Lambe 1996	Sweden	<60	Case-control	Adjusted	76,692	★★★★★★	Low
67	Lambe 1998	Sweden	47.90	Case-control	Adjusted	8,205	★★★★★★	Low
68	Layde 1989	USA	20-55	Case-control	Adjusted	4,599	★★★★★★★★★	High
69	Lee 1992	China	Unspecified	Case-control	Unadjusted	620	★★★★★	Low
70	Leon 1989	UK	16-59	Cohort	Unadjusted	113,263	★★★★★★	Low

ID	Autor	Country	Age mean/range (yr)	Study	adj	Sample size	NOS score	Quality
71	Leon 1995	Sweden	<50	Case-control	Adjusted	28,579	★★★★★★★	High
72	Li 2000	USA	50-64	Case-control	Adjusted	914	★★★★★★	Low
73	Li 2003	USA	65-79	Case-control	Adjusted	1,982	★★★★★★★★	High
74	Li 2007	USA	50-79	Cohort	Adjusted	27,536	★★★★★★★★	High
75	Li 2008	USA	35-64	Case-control	Unadjusted	8,082	★★★★★★	Low
76	Li 2013	USA	20-44	Case-control	Unadjusted	1,962	★★★★★★★★	High
77	Lubin 1981	Canada	56.08	Case-control	Unadjusted	1,403	★★★★★★	Low
78	MacMahon 1982	Estonia	>30	Case-control	Unadjusted	1,056	★★★★★★	Low
79	Madigan 1995	USA	25-74	Cohort	Adjusted	14,407	★★★★★★★	High
80	Magnusson 1999	Sweden	50-74	Case-control	Adjusted	5,279	★★★★★★★	High
81	Mayberry 1992	USA	20-54	Case-control	Unadjusted	8,810	★★★★★★★	High
82	McCredie 1997	Australia	34.06	Case-control	Adjusted	875	★★★★★★★	High
83	McPherson 1987	UK	16-64	Case-control	Adjusted	2,250	★★★★★★	Low
84	Mctiernan 1986	USA	25-54	Case-control	Adjusted	661	★★★★★★★	High
85	Miller 1989	USA	<45	Case-control	Unadjusted	831	★★★★★★	Low
86	Mittendorf 1995	USA	17-74	Case-control	Unadjusted	16,427	★★★★★★★★	High
87	Msolly 2013	Tunisia	49.50	Case-control	Adjusted	800	★★★★★★★	High
88	Nagrani 2015	India	20-69	Case-control	Adjusted	3,152	★★★★★★★	High
89	Negri 1990	Italy	>25	Case-control	Adjusted	2,187	★★★★★★★★	High
90	Negri 1996	Italy	Unspecified	Case-control	Unadjusted	4,375	★★★★★	Low
91	Newcomb 1994	USA	<74	Case-control	Adjusted	14,094	★★★★★★★★	High
92	Newcomb 1995	USA	<75	Case-control	Unadjusted	9,851	★★★★★	Low
93	Newcomb 1996	USA	<75	Case-control	Unadjusted	16,062	★★★★★★	Low
94	Newcomb 1999	USA	50-79	Case-control	Adjusted	7,423	★★★★★★★	High
95	Newcomb 2002	USA	50-79	Case-control	Unadjusted	10,869	★★★★★★	Low
96	Nomura 1984	USA	45-74	Case-control	Adjusted	1,032	★★★★★★★★	High
97	Olsson 1989	Sweden	Unspecified	Case-control	Unadjusted	633	★★★★★	Low
98	Olsson 2002	Sweden	25-65	Cohort	Unadjusted	29,508	★★★★★★	Low
99	Oran 2004	Turkey	Unspecified	Case-control	Adjusted	1,244	★★★★★	Low
100	Paffenbarger 1980	USA	58.12	Case-control	Unadjusted	3,935	★★★★	Low
101	Paul 1986	New Zealand	25-54	Case-control	Unadjusted	1,330	★★★★★★	Low
102	Paul 1990	New Zealand	25-54	Case-control	Unadjusted	2,755	★★★★★★	Low
103	Phipps 2008	USA	68.50	Case-control	Adjusted	2,616	★★★★★★★★	Low
104	Pike 1981	USA	<33	Case-control	Unadjusted	489	★★★★★★★	High
105	Primic-Zakelj 1995	Italy	25.54	Case-control	Adjusted	1,248	★★★★★★★	High
106	Rao 1994	India	44.47	Case-control	Unadjusted	1,400	★★★★★	Low

ID	Autor	Country	Age mean/range (yr)	Study	adj	Sample size	NOS score	Quality
107	Richardson 1998	USA	31.03	Case-control	Unadjusted	573	★★★★★★★	High
108	Romieu 1996	Mexico	20-80	Case-control	Adjusted	1,354	★★★★★★★	High
109	Rosenberg 1984	USA	30-69	Case-control	Unadjusted	2,877	★★★★★★	Low
110	Rosero-Bix 1987	Costa Rica	45.50	Case-control	Adjusted	997	★★★★★★★	High
111	Rossing 1996	USA	50-64	Case-control	Unadjusted	1,029	★★★★★★	Low
112	Sartwell 1977	USA	20-74	Case-control	Unadjusted	651	★★★★★★	Low
113	Schlesselman 1988	USA	20-54	Case-control	Unadjusted	9,254	★★★★★★	Low
114	Siskind 1989	Australia	≤75	Case-control	Unadjusted	1,550	★★★★★	Low
115	Stadel 1985	USA	20-44	Case-control	Unadjusted	4,153	★★★★★★	Low
116	Stadel 1988	USA	20-54	Case-control	Unadjusted	5,591	★★★★★★	Low
117	Stanford 1987	USA	20-54	Case-control	Adjusted	1,026	★★★★★★★	High
118	Stanford 1989	USA	Unspecified	Case-control	Adjusted	4,205	★★★★★★★★	High
119	Stanford 1995	USA	50-64	Case-control	Unadjusted	1,029	★★★★★★	High
120	Storm 1986	Denmark	36.00	Case-control	Adjusted	479	★★★★★★★★	High
121	Talamini 1996	Italy	20-79	Case-control	Adjusted	5,157	★★★★★★★	High
122	Tao 1988	China	47.50	Case-control	Adjusted	994	★★★★★★★★	High
123	Tavani 1997	Italy	20-74	Case-control	Adjusted	5,157	★★★★★★★★	High
124	Thein 1978	Myanmar	Unspecified	Case-control	Unadjusted	593	★★★★★★	Low
125	Titus-Ernstof 1998	USA	50-79	Case-control	Unadjusted	11,587	★★★★★★	Low
126	Toti 1986	Italy	Unspecified	Case-control	Adjusted	3,061	★★★★★★★	High
127	Tovar-Guzmán 2000	Mexico	<75	Case-control	Adjusted	983	★★★★★★★	High
128	Tryggvadottir 2003	Iceland	48.00	Case-control	Adjusted	1,461	★★★★★★★	High
129	Tulinius 1990	Iceland	Unspecified	Cohort	Adjusted	61,040	★★★★★★★	High
130	Ursin 2005	USA	35-64	Case-control	Adjusted	9,257	★★★★★★★★	High
131	Vessy 1975	USA	16-39	Case-control	Unadjusted	734	★★★★★★	Low
132	Wang 1992	China	43.30	Case-control	Adjusted	600	★★★★★★	Low
133	Weiss 1996	USA	38.60	Case-control	Adjusted	3,121	★★★★★★★	High
134	White 1994	USA	21-45	Case-control	Unadjusted	1,708	★★★★★★	Low
135	WHO collaboration	10 nations	Unspecified	Case-control	Adjusted	14,193	★★★★★★★★	High
136	Williams 1990	UK	25-59	Case-control	Adjusted	1,738	★★★★★★★★	High
137	Wingo 1993	Mexico	20-54	Case-control	Unadjusted	9,387	★★★★★	Low
138	Wu 1996	USA	20-55	Case-control	Unadjusted	1,260	★★★★★★	Low
139	Yang 1993	Canada	<75	Case-control	Adjusted	2,043	★★★★★★★	High
140	Yoo 1992	Japan	Unspecified	Case-control	Unadjusted	1,042	★★★★★	Low
141	Yuan 1988	china	20-69	Case-control	Adjusted	1,068	★★★★★★	Low

NOS: The Newcastle-Ottawa Scale

References

1. Adami H, Hansen J, Jung B, Rimsten ÅJ. Age at first birth, parity and risk of breast cancer in a Swedish population. Br J Cancer. 1980; 42:651-8.
2. Adami HO, Bergström R, Lund E, Meirik O. Absence of association between reproductive variables and the risk of breast cancer in young women in Sweden and Norway. Br J Cancer. 1990; 62:122-6.
3. Adami HO, Rimsten A, Stenkvist B, Vegelius J. Reproductive history and risk of breast cancer: a case-control study in an unselected Swedish population. Cancer. 1978; 41:747-57.
4. Albrektsen G, Heuch I, Kvåle G. The short-term and long-term effect of a pregnancy on breast cancer risk: a prospective study of 802,457 parous Norwegian women. Br J Cancer. 1995; 72:480-4.
5. Andrieu N, Goldgar DE, Easton DF, Rookus M, Brohet R, Antoniou AC, et al. Pregnancies, breast-feeding, and breast cancer risk in the International BRCA1/2 Carrier Cohort Study (IBCCS). J Natl Cancer Inst. 2006; 98:535-44.
6. Austin H, Cole P, Wynder E. Breast cancer in black American women. Int J Cancer. 1979; 24:541-4.
7. Babita, Kumar N, Singh M, Malik JS, Kalhan M. Breastfeeding reduces breast cancer risk: a case-control study in north India. Int J Prev Med. 2014; 5:791-5.
8. BAIN C, WILLETT W, ROSNER B, SPEIZER FE, BELANGER C, HENNEKENS CH. Early age at first birth and decreased risk of breast cancer. Am J Epidemiol. 1981; 114:705-9.
9. Barbone F, Filiberti R, Franceschi S, Talamini R, Conti E, Montella M, et al. Socioeconomic status, migration and the risk of breast cancer in Italy. Int J Epidemiol. 1996; 25:479-87.
10. Baron JA, Vessey M, McPherson K, Yeates D. Maternal age and breast cancer risk. J Natl Cancer Inst. 1984; 72:1307-9.
11. Bernstein L, Henderson BE, Hanisch R, Sullivan-Halley J, Ross RK. Physical exercise and reduced risk of breast cancer in young women. J Natl Cancer Inst. 1994; 86:1403-8.
12. Bouchardy C, Lê MG, Hill C. Risk factors for breast cancer according to age at diagnosis in a French case-control study. J Clin Epidemiol. 1990; 43:267-75.
13. Brignone G, Cusimano R, Dardanoni G, Gugliuzza M, Lanzarone F, Scibilia V, et al. A case-control study on breast cancer risk factors in a southern European population. Int J Epidemiol. 1987; 16:356-61.
14. Brinton LA, Daling JR, Liff JM, Schoenberg JB, Malone KE, Stanford JL, et al. Oral contraceptives and breast cancer risk among younger women. J Natl Cancer Inst. 1995; 87:827-35.
15. Brinton LA, Hoover R, Fraumeni JF, Jr. Reproductive factors in the aetiology of breast cancer. Br J Cancer. 1983; 47:757-62.
16. Brinton LA, Hoover R, Szklo M, FRAUMENI Jr JF. Oral contraceptives and breast cancer. Int J Epidemiol. 1982; 11:316-22.
17. Brinton LA, Schairer C, Stanford JL, Hoover RN. Cigarette smoking and breast cancer. Am J Epidemiol. 1986; 123:614-22.
18. Brinton LA, Williams RR, Hoover RN, Stegens NL, Feinleib M, Fraumeni JF, Jr. Breast cancer risk factors among screening program participants. J Natl Cancer Inst. 1979; 62:37-44.
19. Bruzzi P, Green SB, Byar DP, Brinton LA, Schairer C. Estimating the population attributable risk for multiple risk factors using case-control data. Am J Epidemiol. 1985; 122:904-14.
20. Bruzzi P, Negri E, La Vecchia C, Decarli A, Palli D, Parazzini F, et al. Short term increase in risk of breast cancer after full term pregnancy. BMJ. 1988; 297:1096-8.
21. Buchanan AV, Weiss KM, Anderson DE, Chakraborty R, MacNaughton NL. Epidemiology of breast cancer in a Mexican-American population. J Natl Cancer Inst. 1985; 74:1199-206.
22. Butt S, Borgquist S, Anagnostaki L, Landberg G, Manjer J. Parity and age at first childbirth in relation to the risk of different breast cancer subgroups. Int J Cancer. 2009; 125:1926-34.

23. Butt S, Harlid S, Borgquist S, Ivarsson M, Landberg G, Dillner J, et al. Genetic predisposition, parity, age at first childbirth and risk for breast cancer. BMC Res Notes. 2012; 5:414.

24. Byers T, Graham S, Rzepka T, Marshall J. Lactation and breast cancer: evidence for a negative association in premenopausal women. Am J Epidemiol. 1985; 121:664-74.

25. Byrne C, Brinton LA, Haile RW, Schairer C. Heterogeneity of the effect of family history on breast cancer risk. Epidemiology. 1991; 2:276-84.

26. Cancer and Steroid Hormone Study of the Centers for Disease Control and the National Institute of Child Health and Human Development. Oral-contraceptive use and the risk of breast cancer. N Engl J Med. 1986; 315:405-11.

27. Chen C-L, Weiss NS, Newcomb P, Barlow W, White E. Hormone replacement therapy in relation to breast cancer. JAMA. 2002; 287:734-41.

28. Chie W-C, Hsieh C-c, Newcomb PA, Longnecker MP, Mittendorf R, Greenberg ER, et al. Age at any full-term pregnancy and breast cancer risk. Am J Epidemiol. 2000; 151:715-22.

29. Choi NW, Howe GR, Miller AB, Matthews V, Morgan RW, Munan L, et al. An epidemiologic study of breast cancer. Am J Epidemiol. 1978; 107:510-21.

30. Clavel F, Andrieu N, Gairard B, Brémond A, Piana L, Lansac J, et al. Oral contraceptives and breast cancer: a French case-control study. Int J Epidemiol. 1991; 20:32-8.

31. Clavel-Chapelon F, Launoy G, Auquier A, Gairard B, Brémond A, Piana L, et al. Reproductive factors and breast cancer risk. Effect of age at diagnosis. Ann Epidemiol. 1995; 5:315-20.

32. Coogan PF, Rosenberg L, Shapiro S, Hoffman M. Lactation and breast carcinoma risk in a South African population. Cancer. 1999; 86:982-9.

33. Cooper JA, Rohan TE, Cant EL, Horsfall DJ, Tilley WD. Risk factors for breast cancer by oestrogen receptor status: a population-based case-control study. Br J Cancer. 1989; 59:119-25.

34. Craig TJ, Comstock GW, Geiser PB. Epidemiologic comparison of breast cancer patients with early and late onset of malignancy and general population controls. J Natl Cancer Inst. 1974; 53:1577-81.

35. Daling JR, Malone KE, Doody DR, Voigt LF, Bernstein L, Coates RJ, et al. Relation of regimens of combined hormone replacement therapy to lobular, ductal, and other histologic types of breast carcinoma. Cancer. 2002; 95:2455-64.

36. Dolle JM, Daling JR, White E, Brinton LA, Doody DR, Porter PL, et al. Risk factors for triple-negative breast cancer in women under the age of 45 years. Cancer Epidemiol Biomarkers Prev. 2009; 18:1157-66.

37. Enger SM, Ross RK, Henderson B, Bernstein L. Breastfeeding history, pregnancy experience and risk of breast cancer. Br J Cancer. 1997; 76:118-23.

38. Enger SM, Ross RK, Paganini-Hill A, Bernstein L. Breastfeeding experience and breast cancer risk among postmenopausal women. Cancer Epidemiol Biomarkers Prev. 1998; 7:365-9.

39. Ewertz M. Oral contraceptives and breast cancer risk in Denmark. Eur J Cancer. 1992; 28a:1176-81.

40. Ewertz M, Duffy SW. Risk of breast cancer in relation to reproductive factors in Denmark. Br J Cancer. 1988; 58:99-104.

41. Freudenheim JL, Marshall JR, Graham S, Laughlin R, Vena JE, Bandera E, et al. Exposure to breastmilk in infancy and the risk of breast cancer. Epidemiology. 1994; 5:324-31.

42. Furberg H, Newman B, Moorman P, Millikan R. Lactation and breast cancer risk. Int J Epidemiol. 1999; 28:396-402.

43. Gajalakshmi CK, Shanta V. Risk factors for female breast cancer. A hospital-based case-control study in Madras, India. Acta Oncol. 1991; 30:569-74.

44. Gao YT, Shu XO, Dai Q, Potter JD, Brinton LA, Wen W, et al. Association of menstrual and reproductive factors with breast cancer risk: results from the Shanghai Breast Cancer Study. Int J Cancer. 2000; 87:295-300.

45. Gapstur SM, Morrow M, Sellers TA. Hormone replacement therapy and risk of breast cancer with a favorable histology: results of the Iowa Women's Health Study. JAMA. 1999; 281:2091-7.

46. Gapstur SM, Potter JD, Sellers TA, Folsom AR. Increased risk of breast cancer with alcohol consumption in postmenopausal women. Am J Epidemiol. 1992; 136:1221-31.

47. Gilliland FD, Hunt WC, Baumgartner KB, Crumley D, Nicholson CS, Fetherolf J, et al. Reproductive risk factors for breast cancer in Hispanic and non-Hispanic white women: the New Mexico Women's Health Study. Am J Epidemiol. 1998; 148:683-92.

48. Haile RW, Witte JS, Ursin G, Siemiatycki J, Bertolli J, Douglas Thompson W, et al. A case-control study of reproductive variables, alcohol, and smoking in premenopausal bilateral breast cancer. Breast Cancer Res Treat. 1996; 37:49-56.

49. Harris NV, Weiss NS, Francis AM, Polissar L. Breast cancer in relation to patterns of oral contraceptive use. Am J Epidemiol. 1982; 116:643-51.

50. Helmrich SP, Shapiro S, Rosenberg L, Kaufman DW, Slone D, Bain C, et al. Risk factors for breast cancer. Am J Epidemiol. 1983; 117:35-45.

51. Henderson BE, Powell D, Rosario I, Keys C, Hanisch R, Young M, et al. An epidemiologic study of breast cancer. J Natl Cancer Inst. 1974; 53:609-14.

52. Horn J, Åsvold BO, Opdahl S, Tretli S, Vatten LJ. Reproductive factors and the risk of breast cancer in old age: a Norwegian cohort study. Breast Cancer Res Treat. 2013; 139:237-43.

53. Hsieh CC, Chan HW, Lambe M, Ekbom A, Adami HO, Trichopoulos D. Does age at the last birth affect breast cancer risk? Eur J Cancer. 1996; 32a:118-21.

54. Hunt SC, Williams RR, Skolnick MH, Lyon JL, Smart CR. Breast cancer and reproductive history from genealogical data. J Natl Cancer Inst. 1980; 64:1047-53.

55. Innes K, Byers T, Schymura M. Birth characteristics and subsequent risk for breast cancer in very young women. Am J Epidemiol. 2000; 152:1121-8.

56. JICK H, WALKER AM, WATKINS RN, D'EWART DC, HUNTER JR, DANFORD A, et al. Oral contraceptives and breast cancer. Am J Epidemiol. 1980; 112:577-85.

57. Jick H, Walker AM, Watkins RN, D'EWART DC, Hunter JR, DANFORD A, et al. Replacement estrogens and breast cancer. Am J Epidemiol. 1980; 112:586-94.

58. Jick S, Walker A, Stergachis A, Jick H. Oral contraceptives and breast cancer. Br J Cancer. 1989; 59:618-21.

59. Kalache A, Maguire A, Thompson SG. Age at last full-term pregnancy and risk of breast cancer. Lancet. 1993; 341:33-6.

60. Khalis M, Charbotel B, Chajès V, Rinaldi S, Moskal A, Biessy C, et al. Menstrual and reproductive factors and risk of breast cancer: A case-control study in the Fez region, Morocco. PLoS One. 2018; 13:e0191333.

61. Kinlen LJ. Breast cancer and ages at first marriage and first birth: a new hypothesis. Eur J Cancer Prev. 2014; 23:53-7.

62. Kinlen LJ, Gilham C, Ray R, Thomas DB, Peto J. Cohabitation, infection and breast cancer risk. Int J Cancer. 2021; 148:1408-18.

63. Kotsopoulos J, Lubinski J, Lynch HT, Klijn J, Ghadirian P, Neuhausen SL, et al. Age at first birth and the risk of breast cancer in BRCA1 and BRCA2 mutation carriers. Breast Cancer Res Treat. 2007; 105:221-8.

64. Kuru B, Ozaslan C, Ozdemir P, Dinç S, Camlibel M, Alagöl H. Risk factors for breast cancer in Turkish women with early pregnancies and long-lasting lactation--a case-control study. Acta Oncol. 2002; 41:556-61.

65. La Vecchia C, Decarli A, Parazzini F, Gentile A, Negri E, Cecchetti G, et al. General epidemiology of breast cancer in northern Italy. Int J Epidemiol. 1987; 16:347-55.

66. La Vecchia C, Parazzini F, Negri E, Boyle P, Gentile A, Decarli A, et al. Breast cancer and combined oral contraceptives: an Italian case-control study. Eur J Cancer Clin Oncol. 1989; 25:1613-8.

67. Lambe M, Hsieh CC, Chan HW, Ekbom A, Trichopoulos D, Adami HO. Parity, age at first and last birth, and risk of breast cancer: a population-based study in Sweden. Breast Cancer Res Treat. 1996; 38:305-11.

68. Lambe M, Hsieh CC, Tsaih SW, Ekbom A, Trichopoulos D, Adami HO. Parity, age at first birth and the risk of carcinoma in situ of the breast. Int J Cancer. 1998; 77:330-2.

69. Layde PM, Webster LA, Baughman AL, Wingo PA, Rubin GL, Ory HW, et al. The independent associations of parity, age at first full term pregnancy, and duration of breastfeeding with the risk of breast cancer. J Clin Epidemiol. 1989; 42:963-73.

70. Lee HP, Gourley L, Duffy SW, Estève J, Lee J, Day NE. Risk factors for breast cancer by age and menopausal status: a case-control study in Singapore. Cancer Causes Control. 1992; 3:313-22.

71. Leon D. A prospective study of the independent effects of parity and age at first birth on breast cancer incidence in England and Wales. Int J Cancer. 1989; 43:986-91.

72. Leon DA, Carpenter LM, Broeders MJ, Gunnarskog J, Murphy MF. Breast cancer in Swedish women before age 50: evidence of a dual effect of completed pregnancy. Cancer Causes Control. 1995; 6:283-91.

73. Li CI, Beaber EF, Tang MT, Porter PL, Daling JR, Malone KE. Reproductive factors and risk of estrogen receptor positive, triple-negative, and HER2-neu overexpressing breast cancer among women 20-44 years of age. Breast Cancer Res Treat. 2013; 137:579-87.

74. Li CI, Littman AJ, White E. Relationship between age maximum height is attained, age at menarche, and age at first full-term birth and breast cancer risk. Cancer Epidemiol Biomarkers Prev. 2007; 16:2144-9.

75. Li CI, Malone KE, Daling JR, Potter JD, Bernstein L, Marchbanks PA, et al. Timing of menarche and first full-term birth in relation to breast cancer risk. Am J Epidemiol. 2008; 167:230-9.

76. Li CI, Malone KE, Porter PL, Weiss NS, Tang MT, Daling JR. Reproductive and anthropometric factors in relation to the risk of lobular and ductal breast carcinoma among women 65-79 years of age. Int J Cancer. 2003; 107:647-51.

77. Li CI, Stanford JL, Daling JR. Anthropometric variables in relation to risk of breast cancer in middle-aged women. Int J Epidemiol. 2000; 29:208-13.

78. Lubin JH, Burns PE, Blot WJ, Lees AW, May C, Morris LE, et al. Risk factors for breast cancer in women in northern Alberta, Canada, as related to age at diagnosis. J Natl Cancer Inst. 1982; 68:211-7.

79. MacMahon B, Purde M, Cramer D, Hint E. Association of breast cancer risk with age at first and subsequent births: a study in the population of the Estonian Republic. J Natl Cancer Inst. 1982; 69:1035-8.

80. Madigan MP, Ziegler RG, Benichou J, Byrne C, Hoover RN. Proportion of breast cancer cases in the United States explained by well-established risk factors. J Natl Cancer Inst. 1995; 87:1681-5.

81. Magnusson CM, Persson IR, Baron JA, Ekbom A, Bergström R, Adami HO. The role of reproductive factors and use of oral contraceptives in the aetiology of breast cancer in women aged 50 to 74 years. Int J Cancer. 1999; 80:231-6.

82. Mayberry RM, Stoddard-Wright C. Breast cancer risk factors among black women and white women: similarities and differences. Am J Epidemiol. 1992; 136:1445-56.

83. McCredie MR, Dite GS, Giles GG, Hopper JL. Breast cancer in Australian women under the age of 40. Cancer Causes Control. 1998; 9:189-98.

84. McPherson K, Vessey MP, Neil A, Doll R, Jones L, Roberts M. Early oral contraceptive use and breast cancer: results of another case-control study. Br J Cancer. 1987; 56:653-60.

85. McTiernan A, Thomas DB. Evidence for a protective effect of lactation on risk of breast cancer in young women. Results from a case-control study. Am J Epidemiol. 1986; 124:353-8.

86. MILLER DR, ROSENBERG L, KAUFMAN DW, STOLLEY P, WARSHAUER ME, SHAPIRO S. Breast cancer before age 45 and oral contraceptive use: new findings. Am J Epidemiol. 1989; 129:269-80.

87. Mittendorf R, Longnecker MP, Newcomb PA, Dietz AT, Greenberg ER, Bogdan GF, et al. Strenuous physical activity in young adulthood and risk of breast cancer (United States). Cancer Causes Control. 1995; 6:347-53.

88. Msolly A, Gharbi O, Ben Ahmed S. Impact of menstrual and reproductive factors on breast cancer risk in Tunisia: a case-control study. Med Oncol. 2013; 30:480.

89. Nagrani R, Mhatre S, Boffetta P, Rajaraman P, Badwe R, Gupta S, et al. Understanding rural–urban differences in risk factors for breast cancer in an Indian population. Cancer Causes Control. 2016; 27:199-208.

90. Negri E, Braga C, La Vecchia C, Levi F, Talamini R, Franceschi S. Lactation and the risk of breast cancer in an Italian population. Int J Cancer. 1996; 67:161-4.

91. Negri El, La Vecchia C, Duffy S, Bruzzi P, Parazzini F, Day N. Age at first and second births and breast cancer risk in biparous women. Int J Cancer. 1990; 45:428-30.

92. Newcomb PA, Egan KM, Titus-Ernstoff L, Trentham-Dietz A, Greenberg ER, Baron JA, et al. Lactation in relation to postmenopausal breast cancer. Am J Epidemiol. 1999; 150:174-82.

93. Newcomb PA, Longnecker MP, Storer BE, Mittendorf R, Baron J, Clapp RW, et al. Recent oral contraceptive use and risk of breast cancer (United States). Cancer Causes Control. 1996; 7:525-32.

94. Newcomb PA, Storer BE, Longnecker MP, Mittendorf R, Greenberg ER, Clapp RW, et al. Lactation and a reduced risk of premenopausal breast cancer. N Engl J Med. 1994; 330:81-7.

95. Newcomb PA, Titus-Ernstoff L, Egan KM, Trentham-Dietz A, Baron JA, Storer BE, et al. Postmenopausal estrogen and progestin use in relation to breast cancer risk. Cancer Epidemiol Biomarkers Prev. 2002; 11:593-600.

96. Nomura AM, Lee J, Kolonel LN, Hirohata T. Breast cancer in two populations with different levels of risk for the disease. Am J Epidemiol. 1984; 119:496-502.

97. Olsson H, Bladström A. A cohort study of reproductive factors and family history of breast cancer in southern Sweden. Breast Cancer Res Treat. 2002; 76:203-9.

98. Olsson H, Möller TR, Ranstam J. Early oral contraceptive use and breast cancer among premenopausal women: final report from a study in southern Sweden. J Natl Cancer Inst. 1989; 81:1000-4.

99. Oran B, Celik I, Erman M, Baltali E, Zengin N, Demirkazik F, et al. Analysis of menstrual, reproductive, and life-style factors for breast cancer risk in Turkish women: a case-control study. Med Oncol. 2004; 21:31-40.

100. Paffenbarger Jr RS, Kampert JB, Chang H-G. Characteristics that predict risk of breast cancer before and after the menopause. Am J Epidemiol. 1980; 112:258-68.

101. Paul C, Skegg D, Spears G. Oral contraceptives and risk of breast cancer. Int J Cancer. 1990; 46:366-73.

102. Paul C, Skegg DC, Spears GF, Kaldor JM. Oral contraceptives and breast cancer: a national study. Br Med J. 1986; 293:723-6.

103. Phipps AI, Malone KE, Porter PL, Daling JR, Li CI. Reproductive and hormonal risk factors for postmenopausal luminal, HER-2-overexpressing, and triple-negative breast cancer. Cancer. 2008; 113:1521-6.

104. Pike MC, Henderson BE, Casagrande JT, Rosario I, Gray GE. Oral contraceptive use and early abortion as risk factors for breast cancer in young women. Br J Cancer. 1981; 43:72-6.

105. Primic-Žakelj M, Evstifeeva T, Ravnihar B, Boyle P. Breast-cancer risk and oral contraceptive use in slovenian women aged 25 to 54. Int J Cancer. 1995; 62:414-20.

106. Rao DN, Ganesh B, Desai PB. Role of reproductive factors in breast cancer in a low-risk area: a case-control study. Br J Cancer. 1994; 70:129-32.

107. Richardson B, Hulka B, Peck JD, Hughes C, Van den Berg B, Christianson R, et al. Levels of maternal serum alpha-fetoprotein (AFP) in pregnant women and subsequent breast cancer risk. Am J Epidemiol. 1998; 148:719-27.

108. Romieu I, Hernández-Avila M, Lazcano E, Lopez L, Romero-Jaime R. Breast cancer and lactation history in Mexican women. Am J Epidemiol. 1996; 143:543-52.

109. Rosenberg L, Schwingl PJ, Kaufman DW, Miller DR, Helmrich SP, Stolley PD, et al. Breast cancer and cigarette smoking. N Engl J Med. 1984; 310:92-4.

110. Rosero-Bixby L, Oberle MW, Lee NC. Reproductive history and breast cancer in a population of high fertility, Costa Rica, 1984–85. Int J Cancer. 1987; 40:747-54.

111. Rossing MA, Stanford JL, Weiss NS, Habel LA. Oral contraceptive use and risk of breast cancer in middle-aged women. Am J Epidemiol. 1996; 144:161-4.

112. Sartwell PE, Arthes FG, Tonascia JA. Exogenous hormones, reproductive history, and breast cancer. J Natl Cancer Inst. 1977; 59:1589-92.

113. Schlesselman JJ, Stadel BV, Murray P, Lai S. Breast cancer in relation to early use of oral contraceptives. No evidence of a latent effect. JAMA. 1988; 259:1828-33.

114. Sikind V, Schofield F, Rice D, Bain C. Breast cancer and breastfeeding: results from an Australian case-control study. Am J Epidemiol. 1989; 130:229-36.

115. Stadel B, Webster L, Rubin G, Schlesselman J, Wingo P, Cancer, et al. Oral contraceptives and breast cancer in young women. Lancet. 1985; 326:970-3.

116. Stadel BV, Shenghan L, Schlesselman JJ, Murray P. Oral contraceptives and premenopausal breast cancer in nulliparous women. Contraception. 1988; 38:287-99.

117. Stanford JL, Brinton LA, Hoover RN. Oral contraceptives and breast cancer: results from an expanded case-control study. Br J Cancer. 1989; 60:375-81.

118. Stanford JL, Szklo M, Boring CC, Brinton LA, Diamond EA, Greenberg RS, et al. A case-control study of breast cancer stratified by estrogen receptor status. Am J Epidemiol. 1987; 125:184-94.

119. Stanford JL, Weiss NS, Voigt LF, Daling JR, Habel LA, Rossing MA. Combined estrogen and progestin hormone replacement therapy in relation to risk of breast cancer in middle-aged women. Jama. 1995; 274:137-42.

120. Storm HH, Iversen E, Boice JD, Jr. Breast cancer following multiple chest fluoroscopies among tuberculosis patients. A case-control study in Denmark. Acta Radiol Oncol. 1986; 25:233-8.

121. Talamini R, Franceschi S, La Vecchia C, Negri E, Borsa L, Montella M, et al. The role of reproductive and menstrual factors in cancer of the breast before and after menopause. Eur J Cancer. 1996; 32a:303-10.

122. Tao SC, Yu MC, Ross RK, Xiu KW. Risk factors for breast cancer in Chinese women of Beijing. Int J Cancer. 1988; 42:495-8.

123. Tavani A, Braga C, La Vecchia C, Negri E, Russo A, Franceschi S. Attributable risks for breast cancer in Italy: education, family history and reproductive and hormonal factors. Int J Cancer. 1997; 70:159-63.

124. The WHO Collaborative Study of Neoplasia and Steroid Contraceptives. Breast cancer and combined oral contraceptives: results from a multinational study. Br J Cancer. 1990; 61:110-9.

125. Thein H, Thein Maung M. Risk factors of breast cancer in Burma. Int J Cancer. 1978; 21:432-7.

126. Titus-Ernstoff L, Egan KM, Newcomb PA, Baron JA, Stampfer M, Greenberg ER, et al. Exposure to breast milk in infancy and adult breast cancer risk. J Natl Cancer Inst. 1998; 90:921-4.

127. Toti A, Agugiaro S, Amadori D, Buzzi G, Bruzzi P, Buiatti E, et al. Breast cancer risk factors in Italian women: a multicentric case-control study. Tumori Journal. 1986; 72:241-9.

128. Tovar-Guzmán V, Hernández-Girón C, Lazcano-Ponce E, Romieu I, Hernández Avila M. Breast cancer in Mexican women: an epidemiological study with cervical cancer control. Rev Saude Publica. 2000; 34:113-9.

129. Tryggvadottir L, Olafsdottir EJ, Gudlaugsdottir S, Thorlacius S, Jonasson JG, Tulinius H, et al. BRCA2 mutation carriers, reproductive factors and breast cancer risk. Breast Cancer Res. 2003; 5:R121-8.

130. Tulinius H, Sigvaldason H, Hrafnkelsson J, Ólafsdóttir G, Tryggvadóttir L, Sigurðsson K. Reproductive factors and breast cancer risk in Iceland. A second cohort study. Int J Cancer. 1990; 46:972-5.

131. Ursin G, Bernstein L, Lord SJ, Karim R, Deapen D, Press MF, et al. Reproductive factors and subtypes of breast cancer defined by hormone receptor and histology. Br J Cancer. 2005; 93:364-71.

132. Vessey M, Doll R, Jones K. Oral contraceptives and breast cancer: progress report of an epidemiological study. Lancet. 1975; 305:941-4.

133. Wang Q, Ross R, Yu M, Ning J, Henderson B, Kimm H. A case-control study of breast cancer in Tianjin, China. Cancer Epidemiol Biomarkers Prev. 1992; 1:435-9.

134. Weiss HA, Brinton LA, Brogan D, Coates RJ, Gammon MD, Malone KE, et al. Epidemiology of in situ and invasive breast cancer in women aged under 45. Br J Cancer. 1996; 73:1298-305.

135. White E, Malone KE, Weiss NS, Daling JR. Breast cancer among young U.S. women in relation to oral contraceptive use. J Natl Cancer Inst. 1994; 86:505-14.

136. Williams EM, Jones L, Vessey MP, McPherson K. Short term increase in risk of breast cancer associated with full term pregnancy. BMJ. 1990; 300:578-9.

137. Wingo PA, Lee NC, Ory HW, Beral V, Peterson HB, Rhodes P. Age-specific differences in the relationship between oral contraceptive use and breast cancer. Cancer. 1993; 71:1506-17.

138. Wu AH, Ziegler RG, Pike MC, Nomura AM, West DW, Kolonel LN, et al. Menstrual and reproductive factors and risk of breast cancer in Asian-Americans. Br J Cancer. 1996; 73:680-6.

139. Yang CP, Weiss NS, Band PR, Gallagher RP, White E, Daling JR. History of lactation and breast cancer risk. Am J Epidemiol. 1993; 138:1050-6.

140. Yoo KY, Tajima K, Kuroishi T, Hirose K, Yoshida M, Miura S, et al. Independent protective effect of lactation against breast cancer: a case-control study in Japan. Am J Epidemiol. 1992; 135:726-33.

141. Yuan J-M, Yu MC, Ross RK, Gao Y-T, Henderson BE. Risk factors for breast cancer in Chinese women in Shanghai. Cancer Res. 1988; 48:1949-53.

Supplementary Figure 1: Forest plot of case-control studies assessing age at first pregnancy and breast cancer risk

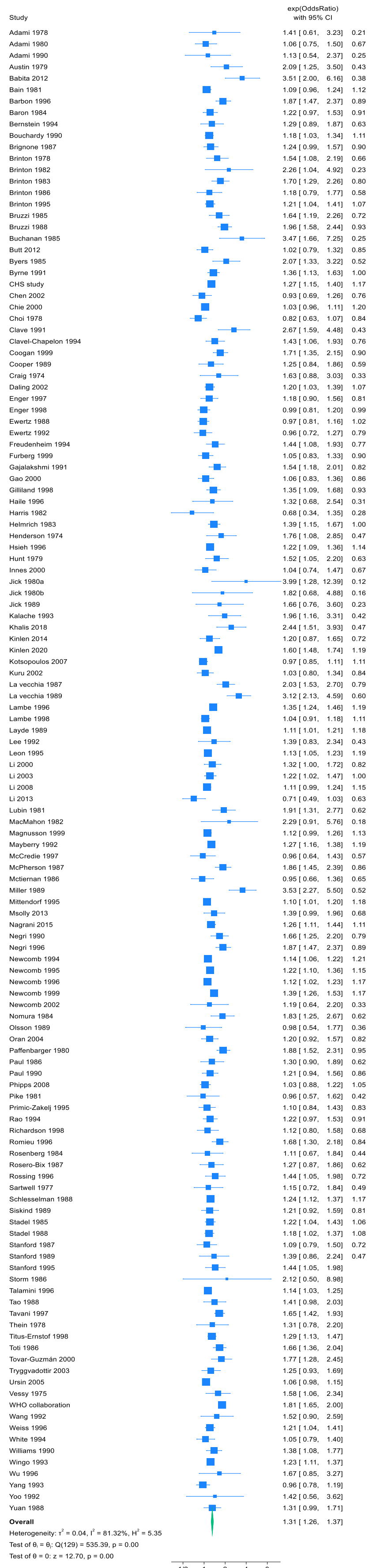

Supplementary Figure 2: Forest plot of case-control studies assessing age at first pregnancy and breast cancer risk following the sensitivity analysis

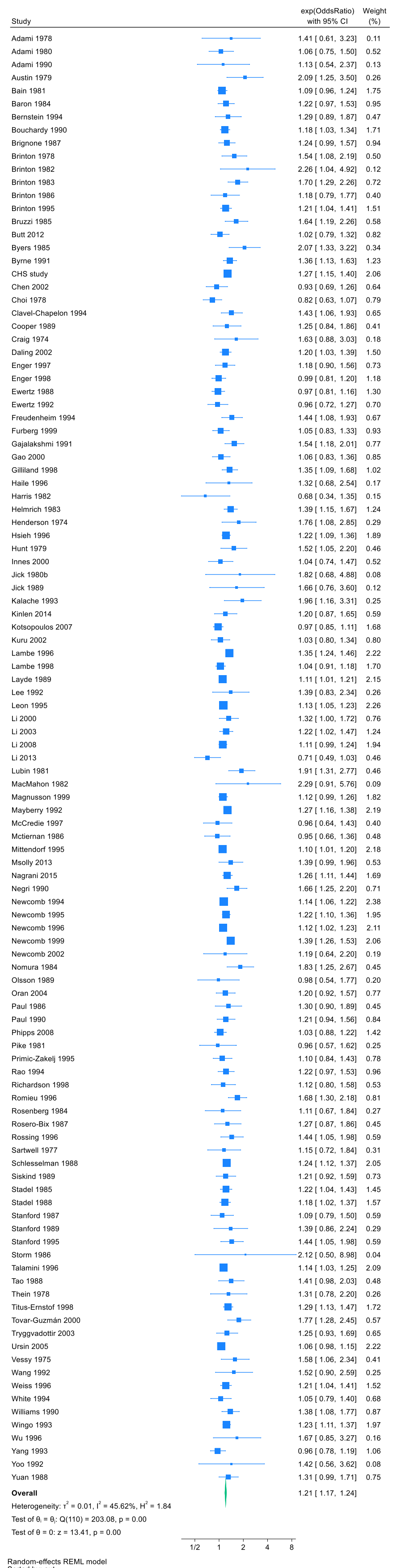

Supplementary Figure 2: Cumulative meta-analysis of case-control studies assessing age at first pregnancy and breast cancer risk, demonstrating sequential and chronological series of meta-analyses with progressive addition of studies to estimate robustness and reliability of the overall association

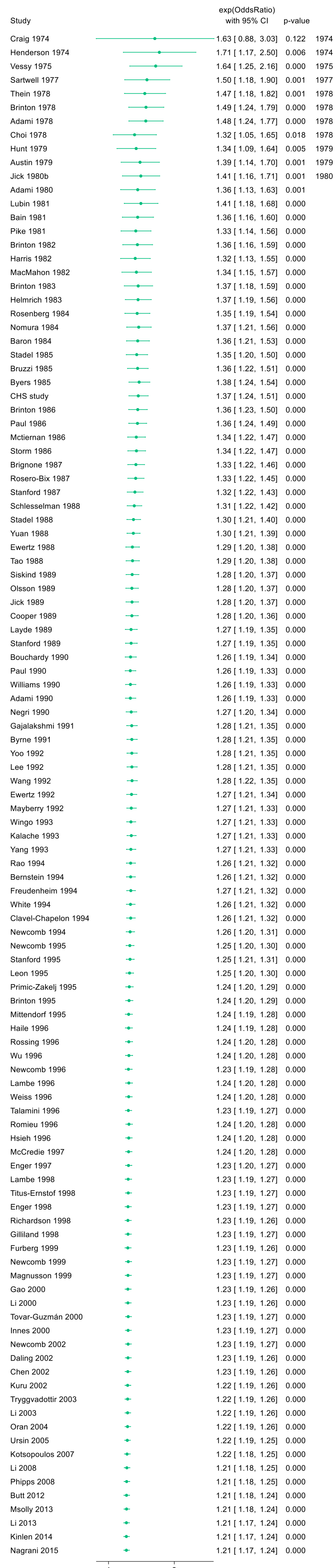

Supplement: Supplementary file 1 [file jrhs-26-e00685-s001.pdf]
